# Interactions between the autochthonous deer ked *Lipoptena cervi* and the alien *L. fortisetosa* (Diptera: Hippoboscidae) ectoparasites of *Cervus elaphus* in Italy: coexistence or competition?

**DOI:** 10.1017/S0031182025000198

**Published:** 2025-03

**Authors:** Laura Stancampiano, Annalisa Andreani, Federica Usai, Patrizia Sacchetti, Maria P. Ponzetta

**Affiliations:** 1 Deparment of Veterinary Medical Sciences, University of Bologna, Bologna, ER, Italy; 2 Department of Agriculture, Food, Environment and Forestry, University of Florence, Florence, TU, Italy

**Keywords:** *Cervus elaphus*, competition, Hippoboscidae, *Lipoptena cervi*, *Lipoptena fortisetosa*, spatial distribution

## Abstract

The autochthonous *Lipoptena cervi* and the allochthonous *Lipoptena fortisetosa* in *Cervus elaphus* in Central/Northern Italy were studied during autumn and winter 2018–2020 in order to evaluate the possible interactions between the two parasite species and the possible influence of geographical parameters on their abundance. This survey could help disentangling whether the coexistence between the two species will be possible or the competitive exclusion of *L. cervi* is to be expected. The results show that *L. cervi* is influenced by host sex and age and is more abundant at higher altitudes, while *L. fortisetosa* is more abundant in lower altitudes and in southern/eastern areas. The interaction between the two species is evident and symmetrical but mild at the component community level, while at the infracommunity level an asymmetric competition has been evidenced by the displacement of *L. cervi* when *L. fortisetosa* is present in the same body location. Geographical clusters of *L. fortisetosa* are evident in plains near urbanized areas, while *L. cervi* distribution appeared more scattered in all the Apennine ridge. Our observations indicate that the two deer ked species not only can coinfect the same host population but also the same host individual, avoiding strong direct interaction and competitive exclusion. All the observed patterns reflect different adaptations to environmental conditions and possible strategies to minimize competition. However, longitudinal surveys are needed to evaluate if the observed pattern is a constant feature or a result of the sampling seasons.

## Graphical abstract

**Figure:**
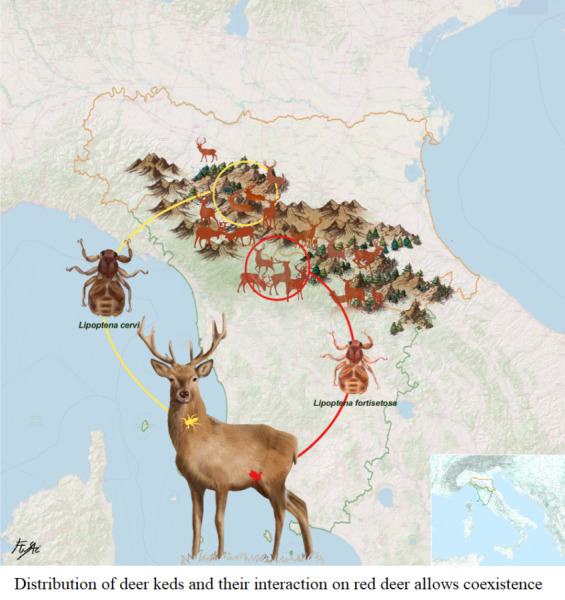

## Introduction

Deer keds (genus *Lipoptena*) are dorsoventrally flattened blood-sucking ectoparasites belonging to the family Hippoboscidae. *Lipoptena cervi* is a native species in Italy, reported in central and northern regions, and distributed across more than 20 European countries with a gradual expansion northward (Andreani *et al*., 2021). Until a few years ago, *L. cervi* was the only deer ked known in Italy, but the recent report of *Lipoptena fortisetosa* (Andreani *et al*., 2019), a species native to Japan, has changed the scenario highlighting the coexistence of these two congeneric species, both living in the same Italian area (the Tuscan-Emilian Apennines) and sharing the same hosts. Nowadays, *L. fortisetosa* is present in many European countries, with a range extending from Estonia as the northernmost limit of distribution, to Switzerland and Italy as the westernmost and southernmost limits (Kurina *et al*., 2019).

Both *L. cervi* and *L. fortisetosa* are blood-feeding ectoparasites that spend their adult life on wild ungulates, especially cervids, where they mate. The female produces a single larva at a time retaining it inside the body until it is ready to pupate; the newborn prepupa immediately moults forming the puparium and falls to the ground. Each female generates several larvae over its lifespan. The pupal stage can last several months, since the species often overwinter in this stage before emerging as winged adult that immediately begins searching for a suitable host. Once they settled on a suitable host, the winged adults will lose their wings and remain closely associated with the selected individual host for their entire life, which can last several months (Haarløv, 1964).

It is known that *L. cervi* is a univoltine species, with adults emerging in late summer and autumn; this species is able to resist to very low temperatures and prefers enclosed shady environments with moist and soft substrates suitable for protecting the pupae (Haarløv, 1964). Although similar, *L. fortisetos*a might exhibit different seasonal pattern and environmentalpreferences. Kowal *et al*. (2016), for example, stated that this species is probably multivoltine. The two species, therefore, seem to have similarities that could induce competitive interactions but also different strategies that can allow their coexistence.

In Italy, a preliminary survey about the presence of *L. cervi* and *L. fortisetosa* in cervids has shown that both species have a preference for the red deer, *Cervus elaphus*, rather than other cervids (Andreani *et al*., 2021). The first aim of the present paper is to specifically analyse the parasitological data, already described in Andreani *et al*. (2021), in the main host, considering the influence of geographical parameters (i.e. the stationary variables not included in the analyses of the previous paper) on the abundance of the two species; the second aim is to evaluate the possible interaction between the two *Lipoptena* at the infracommunity (i.e. in individual hosts) and component community (i.e. in the host population) level, in order to understand if coexistence will be possible or the competitive exclusion of *L. cervi* is to be expected. From a broader ecological perspective the results should be useful for the comprehension of the complex dynamics between alien and native species.

## Material and methods

**Figure 1.**
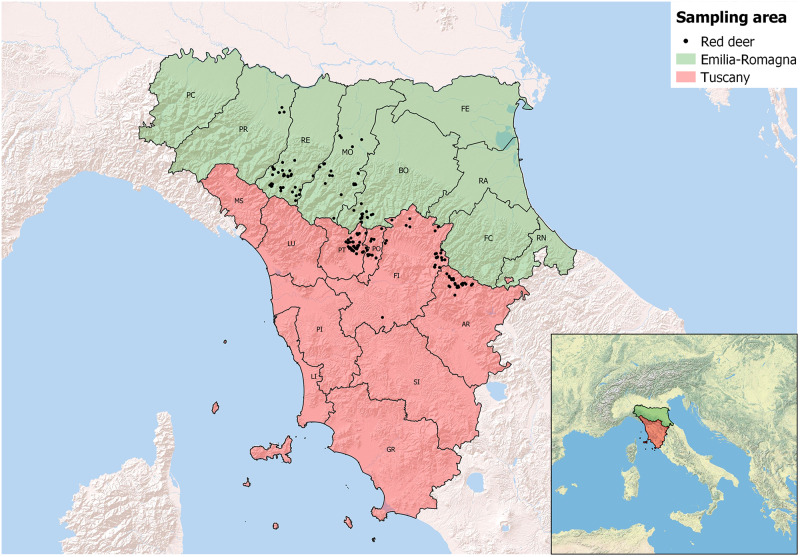
Geographical distribution of the red deer examined in the present survey (administrative districts labelled by their respective acronyms).

Deer keds were collected from red deer hunted in two neighbouring Regions (Emilia-Romagna and Tuscany) of Northern and Central Italy during the autumn and winter seasons from 2018 to 2020 (Figure 1). Ectoparasites were counted separately from the neck and the groin body areas of each red deer, and subsequently identified following taxonomical keys and morphological descriptions. All the procedures regarding sampling and insect identification have been already described in detail in Andreani *et al*. (2021).

### Statistical analyses

Statistical analyses were performed using STATA 12.1. Descriptive and univariate statistics (Chi-square test for categorical variables and t-Student test for continuous variables) were used to describe the dataset while performing preliminary data analysis. In order to simplify the interpretation of the results, continuous variables (altitude, longitude and latitude) were standardized prior to the multivariate analyses described below.

Generalized linear models, specifically negative binomial regressions, were used to evaluate the relationship of parasite abundances (defined according to Margolis *et al*., 1982) with possible covariates related to host (sex, age-class), environment (altitude, longitude, latitude) and administrative boundary (Tuscany or Emilia-Romagna Region).

Negative binomial regressions were also used to highlight competitive interactions between the two parasite species, both at the host population level (i.e. abundance of each parasite species in relation to the other) and at the single host level (i.e. the relation between the abundance of one species in groin and neck and the abundance of the other species in the same body areas). According to Stancampiano *et al*. (2010), the inclusion of the total abundance of the parasite species as a dependent variable in these last models allows us to evaluate if the presence of a *Lipoptena* species in a particular site, while holding constant its total number, was influenced by the presence of the other species in the same site.

The most parsimonious negative binomial models, built via backward eliminations, were selected comparing them to the full models using Likelihood-ratio test and Akaike Information Criterion.

The IRR (Incidence Rate Ratio) of the Negative Binomial regression models is to be interpreted as the natural logarithm of the ratio of expected counts (https://stats.oarc.ucla.edu/stata/output/negative-binomial-regression/).

### Spatial analyses

Latitude and longitude of the location where each deer was hunted were provided by hunters and obtained by the local hunting agency. Spatial analysis was performed importing the geographic coordinates of each sampling location into QGIS (version 3.16, Hannover); through the same software the site altitude was derived. A shapefile was created containing the point locations of all sampling sites. The numbers of *L. cervi* and *L. fortisetosa* collected were associated with each point, creating an attribute table that included all relevant variables for subsequent analysis. The spatial reference system used was WGS 84.

To assess the spatial autocorrelation of parasite distribution, the Local Moran’s Index (I) (Anselin, 1995) was calculated; it indicates whether and to what extent the value of a variable in one location is similar to the values in nearby locations. This analysis was performed using the Hotspot Analysis tool available in the QGIS software (Oxoli *et al*., 2017). A spatial weights matrix was defined using a fixed distance band to ensure at least one neighbour. Positive values of Local Moran’s I indicate clusters of similar values (either high or low), while negative values indicate spatial outliers. Spatial clusters include high–high clusters (HH – high values in a high value neighbourhood) and low–low clusters (LL – low values in a low value neighbourhood). Meanwhile, a negative Local Moran’s I value implies a potential spatial outlier, which is different from the values of its surrounding locations. Spatial outliers include high–low (HL – a high value in a low value neighbourhood) and low–high (LH – a low value in a high value neighbourhood) outliers. A value close to zero indicates a random distribution, without an evident spatial pattern (Fu *et al*., 2014).

## Results

### Preliminary analyses

Sex and age-class of the examined red deer, grouped according to their Region of sampling, are reported in Table 1. No significant differences regarding sex and age-class of the examined deer were highlighted between Emilia-Romagna and Tuscany (Chi-square test, p-values: 0.55 and 0.21, respectively).

**Table 1.** Number of red deer examined in each region stratified by sex and age-class

Region	Red deer sex			Red deer age-class		
	Male	Female		Fawn	Subadult	Adult
Emilia-Romagna (#75)	53.3% (#40)	46.7% (#35)		21.3% (#16)	26.7% (#20)	52.0% (#39)
Tuscany (#102)	57.8% (#59)	42.2% (#43)		18.6% (#19)	39.2% (#40)	42.2% (#43)
Total (#177)	55.9% (#99)	44.1% (#78)		19.8% (#35)	33.9% (#60)	46.3% (#82)

Altitude ranged from 35.5 to 1259.4 m above sea level (average 581.4 m); the mean altitude did not significantly differ between Regions (t-Student test, p-value = 0.13).

From the 177 examined red deer, 21386 hippoboscids were collected; 4335 flies have been identified as *L. cervi* and 17051 as *L. fortisetosa*. Overall prevalence, abundance, minimum and maximum of the two ectoparasite species in both the examined host body areas are reported in Table 2, where it is possible to observe that *L. cervi* was more abundant in the neck, while *L. fortisetosa* in the groin area.

**Table 2.** Prevalence %, abundance and standard deviation (s.d.), minimum and maximum (min–max) of *Lipoptena cervi* and *Lipoptena fortisetosa* collected from neck and groin areas of 177 red deer examined in the present survey

	Prevalence	Abundance (s.d.)	Min–max
*L. Cervi*			
Total	76.3%	24.5 (52.1)	0–398
Groin	52.0%	4.2 (10.3)	0–83
Neck	65.5%	20.2 (45.0)	0–315
*L. fortisetosa*			
Total	71.7%	96.3 (239.0)	0–1844
Groin	68.4%	72.6 (172.7)	0–1149
Neck	34.5%	23.7 (88.5)	0–695

**Figure 2.**
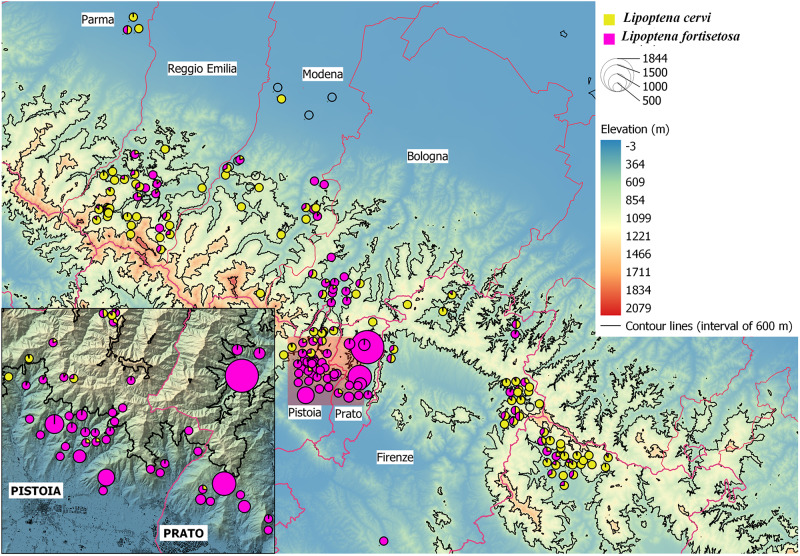
Distribution of *Lipoptena fortisetosa* and *Lipoptena cervi* in the sampling area. Each piechart shows the relative proportion of the two species, highlighting the coexistence of the parasite species in the studied territory. The inset map shows a zoomed view near Prato and Pistoia. Digital elevation model provided by TINITALY/1.1 (grid resolution: 10 m, Tarquini *et al*., 2023).

Figure 2 shows the distribution and the relative intensities of *L. cervi* and *L. fortisetosa* in the sampling area.

The prevalence of *L. cervi* did not significantly differ between Emilia-Romagna and Tuscany Regions (chi-square test, p-value = 0.521) while *L. fortisetosa* was significantly more prevalent in Tuscany (83.3%) than in Emilia-Romagna (56%) (chi-square test, p-value < 0.001).

Out of 177 examined red deer, 10 had no *Lipoptena* spp., 95 had both the species and 72 had just one of the two species (40 hosts with *L. cervi* only and 32 with *L. fortisetosa* only); Chi-square test for independence did not highlight any significant relationship between the presence of the two species (p-value = 0.464), meaning that the frequency of co-occurrence did not deviate from randomness.

**Figure 3.**
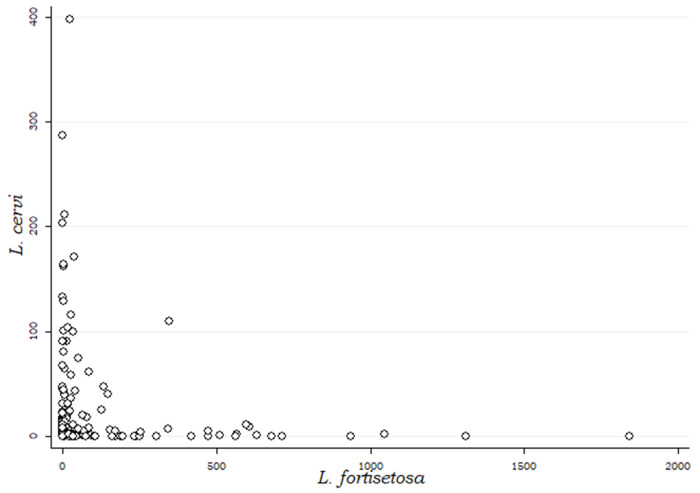
Scatterplot showing the relationship between the intensities of infection of *Lipoptena cervi* and *Lipoptena fortisetosa* in the examined red deer.

Regarding the relationship between the intensity of infection of the two species, a preliminary graphic evaluation is shown in Figure 3: the shape of the graphic clearly indicates that the higher intensities of infections of *L. fortisetosa* are observable in animals with very low intensities of *L. cervi*, and *vice versa*.

**Figure 4.**
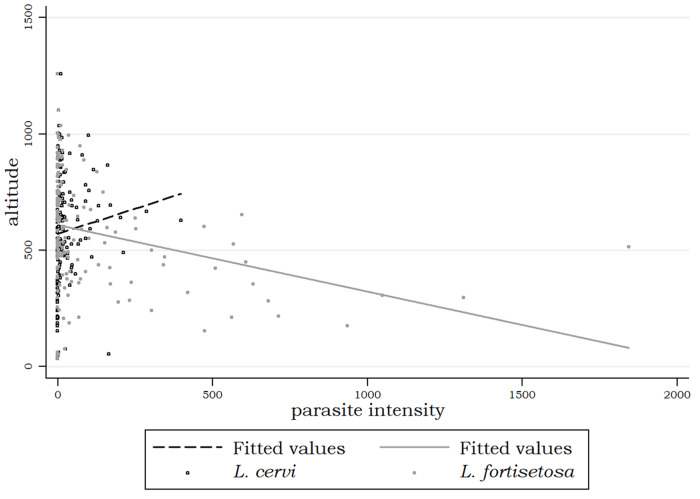
Scatterplots and linear fitted values showing the relationship between each *Lipoptena* species and altitude.

In Figure 4 is reported a preliminary graphic evaluation of the relationship between each parasite species and altitude. Starting from 600 m above sea level, where both species tend to have similar abundances, *L. cervi* increases its abundance at higher altitudes while *L. fortisetosa* increases its abundance at lower altitudes.

### Multivariate analyses: parasite abundance at the host population level

The abundance of *L. cervi* (Table 3) is clearly influenced by sex and age class of the hosts, being this parasite species more abundant in subadults and adults than in fawns (IRR > 1) and less abundant in females than in males (IRR < 1), while, among stationary variables, only altitude exert a positive effect (IRR > 1); the abundance of *L. fortisetosa* has only a slight (although significant at p < 0.001) negative influence (IRR very close to 1).

**Table 3.** Negative binomial regression most parsimonious model having the abundance of *Lipoptena cervi* as dependent variable; ref. is the reference value for categorical covariates. IRR: incidence rate ratio

Abundance of *L. cervi*	IRR	p-value	95% Confidence interval.
Sex (ref. = male)			
Female	0.347	0.001	0.189–0.638
Age (ref. = fawn)			
Subadult	11.104	0.000	5.220–23.621
Adult	10.380	0.000	5.277–20.418
Standardized altitude	1.382	0.032	1.029–1.857
*L. fortisetosa* abundance	0.997	0.000	0.996–0.998
Constant	4.186	0.000	2.191–7.994

The abundance of *L. fortisetosa* (Table 4) is mainly influenced by stationary variables, being less abundant at higher altitudes, higher latitudes (northernmost areas) and higher longitudes (easternmost areas); the abundance of *L. cervi* has only a slight (although significant at p < 0.05) negative influence on *L. fortisetosa* abundance (IRR very close to 1).

**Table 4.** Negative binomial regression most parsimonious model having the abundance of *Lipoptena fortisetosa* as dependent variable. IRR: incidence rate ratio

Abundance of *L. fortisetosa*	IRR	p-value	95% Confidence interval.
Standardized altitude	0.412	0.000	0.287–0.592
Standardized latitude	0.132	0.000	0.079–0.220
Standardized longitude	0.265	0.000	0.132–0.531
*L. cervi* abundance	0.994	0.039	0.989–0.999
Constant	45.44	0.000	33.216–62.185

### Multivariate analyses: interaction at the host individual level

The analysis of interspecific competition between *L. cervi* and *L. fortisetosa* at the individual host level evidences asymmetrical interaction between species: the number of *L. cervi* in groin, given the total number of *L. cervi*, is negatively influenced by the number of *L. fortisetosa* in the same body area (Table 5); in the same way, the number of *L. cervi* in neck, given the total number of *L. cervi*, is negatively influenced by the number of *L. fortisetosa* in the same body area (Table 6). On the contrary, the number of *L. fortisetosa* in groin or in neck is not significantly influenced by the presence of *L. cervi* in the same body areas (Tables 7 and 8).

**Table 5.** Negative binomial regression most parsimonious model having the abundance of *Lipoptena cervi* in the groin region as dependent variable. IRR: incidence rate ratio

*L. cervi* in groin	IRR	p-value	95% Confidence interval
*L. cervi* (total)	1.019	0.000	1.012–1.025
*L. fortisetosa* in groin	0.997	0.008	0.995–0.999
Constant	1.761	0.001	1.274–2.434

**Table 6.** Negative binomial regression most parsimonious model having the abundance of *Lipoptena cervi* in the neck region as dependent variable; ref. is the reference value for categorical covariates. IRR: incidence rate ratio

*L. cervi* in neck	IRR	p-value	95% Confidence interval.
Age (ref. = fawn)			
subadult	3.793	0.000	1.990–7.233
adult	4.147	0.000	2.269–7.581
Standardized altitude	1.384	0.006	1.096–1.747
*L. cervi* total	1.031	0.000	1.024–1.038
*L. fortisetosa* in neck	0.995	0.009	0.992–0.999
Constant	0.953	0.855	0.567–1.600

**Table 7.** Negative binomial regression most parsimonious model having the abundance of *Lipoptena fortisetosa* in the groin region as dependent variable. IRR: incidence rate ratio

*L. fortisetosa* in groin	IRR	p-value	95% Confidence interval.
Standardized longitude	0.645	0.006	0.471–0.883
*L. fortisetosa* total	1.008	0.000	1.005–1.010
Constant	9.804	0.000	6.894–13.491

**Table 8.** Negative binomial regression most parsimonious model having the abundance of *Lipoptena fortisetosa* in the neck region as dependent variable. IRR: incidence rate ratio

*L. fortisetosa* in neck	IRR	p-value	95% Confidence interval.
Standardized altitude	0.515	0.001	0.347–0.763
Standardized longitude	0.566	0.009	0.370–0.867
*L. fortisetosa* total	1.009	0.000	1.006–1.011
*L. cervi* in neck	0.990	0.060	0.980–1.000
Constant	0.938	0.798	0.575–1.530

### Spatial analysis (Local Moran’s I)

**Figure 5.**
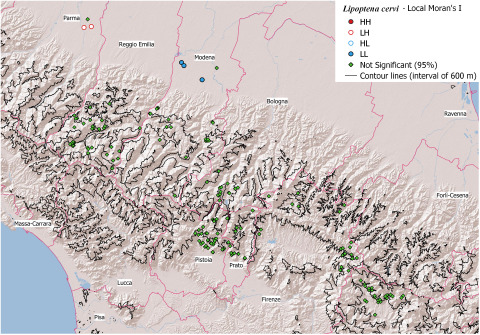
Local Moran’s I cluster/outlier map of *Lipoptena cervi*. The legend indicates the categories of spatial pattern: HH (high–high), LH (low–high), HL (high–low) and LL (low–low) and not significant points.

**Figure 6.**
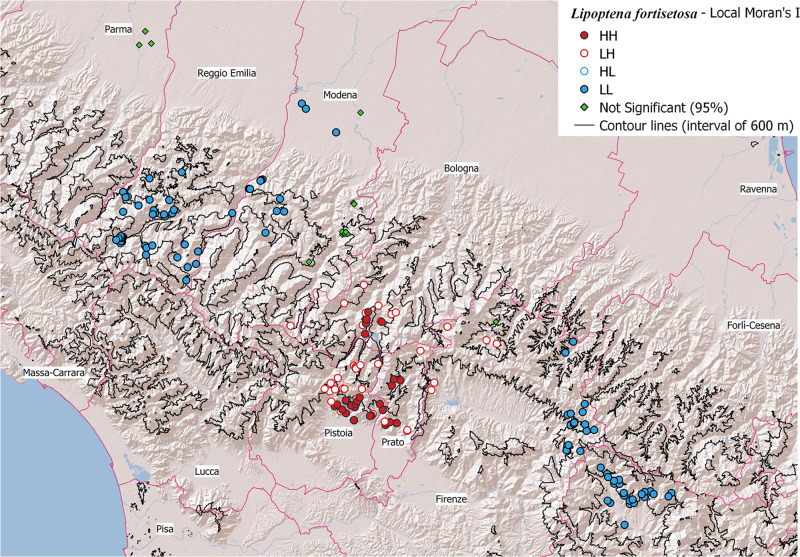
Local Moran’s I cluster/outlier map for *Lipoptena fortisetosa*. The legend indicates the categories of spatial pattern HH (high–high), LH (low–high), HL (high–low), LL (low–low) and not significant points.

The results of the spatial analysis are showed in Figures 5 and 6 for *L. cervi* and *L. fortisetosa* respectively. These figures show the cluster-outlier maps, where sampling locations are coded based on the significance and direction of spatial autocorrelation allowing for easy identification of HH, HL, LH and LL categories.

*Lipoptena fortisetosa* shows clearly separated clusters: one HH cluster around the cities of Pistoia and Prato; two clusters LL located in the areas of higher altitudes; in addition, LH areas of negative spatial autocorrelation are observable around the HH cluster. *Lipoptena cervi*, instead, shows only few points of positive autocorrelation LL and negative autocorrelation LH, while throughout most of the area no significant spatial autocorrelation appears.

## Discussion

This survey has revealed interesting patterns and different geographical preferences of the two fly species in Northern and Central Italy, thanks to the inclusion of stationary variables (altitude, latitude and longitude) to the preliminarily described data in Andreani *et al*. (2021). *Lipoptena cervi* and *L. fortisetosa* appear quite geographically separated: *L. fortisetosa* is more abundant at altitudes below 600 m and is particularly concentrated around the cities of Pistoia and Prato, while *L. cervi* shows a clear preference for altitudes above 600 m, with a distribution along the Tuscan-Emilian Apennine ridge (Figures 2 and 4).

The geographical separation of the two species is highlighted also by the Moran’s Index (I). As shown in Figure 6, *L. fortisetosa* exhibits pronounced positive spatial autocorrelation with infrapopulations concentrated in distinct areas (clusters HH of high intensities values) and areas with very low abundances (clusters LL of low intensities values). High abundance values tend to prevail at lower altitudes, suggesting that the environmental conditions in plain areas are probably more conducive to its survival and reproduction. Conversely, spatial proximity of dissimilar values (LH spatial pattern, indicating negative spatial autocorrelation) indicates that even in some favourable locations individual *L. fortisetosa* intensities are lower than the local mean; it is consistent with the typical aggregated parasite distribution, where the variance is larger than the mean and most hosts have parasite burdens lower than the mean (Crofton, 1971), distribution already tested in Andreani *et al*. (2021).

In contrast, *L. cervi* is predominantly distributed randomly, without a clear spatial pattern; only a few points of positive autocorrelation LL are present in the northern area near Modena city (Figure 5).

While the spatial aggregation of *L. fortisetosa* may indicate that this species benefits from conditions that are patchily distributed at lower altitudes, the observation that *L. cervi* is randomly distributed, although it prefers higher altitudes, could be explained by the conditions favouring red deer in Italy, such as forests far from cultivation and urban areas (Mattioli *et al*., 2001) being the same that favour *L. cervi*. Higher altitudes might offer climatic or vegetative conditions that are tolerable for *L. cervi* but less favourable for *L. fortisetosa*.

Data about the bioecology of *L. cervi* are reasonably accessible, while *L. fortisetosa* have been mainly studied in Japan and most papers are published in languages other than English (see for example Nakayama, 2007; Sonobe, 1979). However, the differences reported in the available literature between the two species as regards latitude, temperature, seasonality and preferred habitats are consistent with the results of the present paper.

*Lipoptena cervi* is reported in Northern Europe even at very high latitudes (Härkönen *et al*., 2010; Mysterud *et al*., 2016) and it is expanding its range in the northern region of Europe (Välimäki *et al*., 2010). It is also reported in the North-eastern United States (Samuel *et al*., 2012) while the related species *Lipoptena mazamae* is spread in the Southern United States (Poh *et al*., 2022). Conversely, *L. fortisetosa* is usually reported at lower latitudes (Choi *et al*., 2013; Kurina *et al*., 2019).

Regarding temperature, the environmental stages (pupae and flying adults) of *L. cervi* demonstrate a marked tolerance to cold (Haarløv, 1964; Härkönen *et al*., 2010, 2012; Nieminen *et al*., 2012) while no specific data are available for *L. fortisetosa*, although Gałęcki *et al*. (2020) found a positive correlation between winged adult abundance and temperature. Also, the favourite habitat is well defined only for *L. cervi*, that prefers cooler shadier woody areas than open grasslands (Haarløv, 1964). Our results indicate that *L. fortisetosa*, at least in Northern and Central Italy, has different habitat preferences compared to *L. cervi*, since a large cluster with high abundances near anthropic areas have been observed (Figure 6).

The seasonal pattern of the two *Lipoptena* species is certainly different. Haarløv (1964) reported that in northern Europe the winged stages of *L. cervi* have a peak around September-October while the wingless stage on the hosts are at its minimum in the summer; the same author states that the number of generations is limited to one per year, being *L. cervi* a univoltine species. The peak of winged adults is confirmed by Mysterud *et al*. (2016) in Norway, where the authors relate the seasonal pattern more to the temperature drop than to the photoperiod. The seasonal pattern of *L. cervi* has not been studied in Italy, but it is reasonable that in South Europe the peak of winged adults, and therefore of wingless forms on the host, will be delayed because of higher temperature than Northern European regions. *Lipoptena fortisetosa* was suspected to be multivoltine by Mogi (1975), that, in Japan, observed the first emergence of winged adults of this species in summer. Sonobe (1979) confirmed the suspect that this species has more than one generation per year; however, in our best knowledge no longitudinal study of the life cycle of *L. fortisetosa* have been published up to now.

Different preferences for altitude, temperature and season of *L. cervi* and *L. fortisetosa* account for their possible coexistence in the same deer population, leaving minimal space for their competition; however, the presence of overlapping niches, especially around 600 m of altitude and probably during intermediate seasons, is consistent with the residual negative relation revealed by the multiple regression in the present survey (Tables 5 and 6). Mixed infections sustained by deer keds of different *Lipoptena* species in the same host population and on the same individual deer have long been considered unusual (Mogi, 1975; Kowal *et al*., 2016). Indeed, most reported infections are monospecific, with different deer ked species parasitizing separate populations. A paradigmatic example is the strong geographical separation between *L. mazamae* and *L. cervi* in the eastern United States reported by Poh *et al*. (2022). In particular, -in host populations different from the one examined in the present paper and in Andreani *et al*. (2021)- *L. cervi* has been reported in mixed infection only by Klepeckienè *et al*. (2020), being the coexistent species *L. fortisetosa*. It is interesting to note that in Japan, where *L. fortisetosa* and *L. sikae* are both reported since 1975, mixed infections appears more common (Mogi, 1975; Sonobe, 1979; Yamauchi and Nakayama, 2006), suggesting that it is a characteristic of *L. fortisetosa* (maybe thanks to its biology different from that of other *Lipoptena* species) to be able to coexist on the same hosts with other congeneric species.

The coinfection observed in the present survey allowed the study of the direct interaction of the two species at the individual host level. Our models show an asymmetric interaction between the two species, with *L. fortisetosa* preferentially localizing in the groin area and *L. cervi* partially moving to the neck when high densities of *L. fortisetosa* are present in the groin. In literature, *L. cervi* localization on the host body appears quite similar when comparing anterior and posterior areas. According to Haarløv (1964) this species was found 25% in the neck and 23% in the groin; Poh *et al*. (2022) observed that the abundances of this species are similar in anterior and posterior parts of the body, differently from *L. mazamae*. Unfortunately, no data are available about *L. fortisetosa*. Our results suggest an asymmetric interaction between *L. fortisetosa* and *L. cervi*, with the latter preferring the anterior part of the body in the presence of numerous *L. fortisetosa* in the groin. At first glance, this result appears quite surprising as in other occasion of asymmetric competition for spatial niche it is the smaller parasite to move far from the bigger one (Stancampiano *et al*., 2010), while in this case the allochthonous *L. fortisetos*a is smaller than the autochthonous *L. cervi* (Andreani *et al*., 2019). However, the earlier emergence of adult *L. fortisetosa* in summer could give it an advantage in the choice of the preferred body site over the delayed *L. cervi* which, on the other hand, can coexist on the same host thanks to its indifference for neck or groin. Our observations indicate that the two deer ked species not only can coinfect the same host population but also the same host individual, avoiding strong direct interaction and competitive exclusion.

All the observed patterns reflect different adaptations to environmental conditions and possible strategies to minimize competition. The coexistence in the overlapping altitudinal range suggests an ecological balance where both species can survive without a clear competitive advantage for either, possibly due to a combination of environmental factors that do not particularly favour either species. The higher abundance of the allochthonous *L. fortisetosa*, reported in the previous study by Andreani *et al*. (2021), seemed to reveal a possible future competitive exclusion of *L. cervi*. The present analysis does not support this supposition; however, longitudinal surveys are needed to evaluate if the abundance ratio in favour of *L. fortisetosa* is a constant feature or a result of the period of the year (autumn and winter), which cannot be excluded considering the different seasonal pattern of the two species reported above.
